# Thiopurine methyltransferase and treatment outcome in the UK acute lymphoblastic leukaemia trial ALL2003

**DOI:** 10.1111/bjh.13469

**Published:** 2015-05-05

**Authors:** Lynne Lennard, Cher S Cartwright, Rachel Wade, Ajay Vora

**Affiliations:** 1 Department of Human Metabolism, University of SheffieldSheffield, UK; 2 Clinical Trials Service UnitOxford, UK; 3 Department of Paediatric Haematology, Children's HospitalSheffield, UK

**Keywords:** thiopurine methyltransferase, mercaptopurine, acute lymphoblastic leukaemia, minimal residual disease

## Abstract

The influence of thiopurine methyltransferase (*TPMT*) genotype on treatment outcome was investigated in the United Kingdom childhood acute lymphoblastic leukaemia trial ALL2003, a trial in which treatment intensity was adjusted based on minimal residual disease (MRD). *TPMT* genotype was measured in 2387 patients (76% of trial entrants): 2190 were homozygous wild-type, 189 were heterozygous for low activity *TPMT* alleles (166 TPMT*1/*3A, 19 TPMT*1/*3C, 3 TPMT*1/*2 and 1 TPMT*1/*9) and 8 were TPMT deficient. In contrast to the preceding trial ALL97, there was no difference in event-free survival (EFS) between the *TPMT* genotypes. The 5-year EFS for heterozygous TPMT*1/*3A patients was the same in both trials (88%), but for the homozygous wild-type TPMT*1/*1 patients, EFS improved from 80% in ALL97% to 88% in ALL2003. Importantly, the unexplained worse outcome for heterozygous TPMT*1/*3C patients observed in ALL97 (5-year EFS 53%) was not seen in ALL2003 (5-year EFS 94%). In a multivariate Cox regression analysis the only significant factor affecting EFS was MRD status (hazard ratio for high-risk MRD patients 4·22, 95% confidence interval 2·97–5·99, *P* < 0·0001). In conclusion, refinements in risk stratification and treatment have reduced the influence of *TPMT* genotype on treatment outcome in a contemporary protocol.

The thiopurine drug mercaptopurine has been an integral component of the maintenance chemotherapy within protocols for childhood acute lymphoblastic leukaemia (ALL) for many decades (Burchenal *et al*, 1953); disease relapse is significantly reduced by long-term maintenance (Richards *et al*, 1996; Schrappe *et al*, 2000). The precise mechanism of action of mercaptopurine in the control and eradication of residual leukaemia cells is open to debate (Gale & Butturini, 1991), but the thioguanine nucleotide (TGN) active metabolites can exert their effects in a number of ways. The TGNs can induce apoptotic cell death by inhibition of intracellular signalling pathways (Tiede *et al*, 2003; Poppe *et al*, 2006; Bourgine *et al*, 2011). The TGNs can also inhibit DNA methylation and so promote cytotoxicity (Hogarth *et al*, 2008) whist cytotoxicity can be triggered by the direct incorporation of drug-derived TGN metabolites into DNA (Tidd & Paterson, 1974; Karran, 2006).

The polymorphic enzyme thiopurine methyltransferase (TPMT) regulates intracellular TGN metabolite production from the mercaptopurine pro-drug; there is an inverse relationship between TPMT activity and TGN production (Lennard *et al*, 1990, 2013; Relling *et al*, 1999a). TPMT deficiency (homozygous for a variant low activity allele, 1 in 300 subjects) is associated with an excess production of TGN metabolites and life-threatening bone-marrow toxicity if such patients are treated with standard doses of thiopurine drugs (Weinshilboum & Sladek, 1980; Lennard *et al*, 1989; Evans *et al*, 1991; McBride *et al*, 2000). *TPMT* heterozygotes (intermediate activity, 11% of subjects) accumulate higher TGN concentrations than those subjects with a wild-type genotype and the former are more sensitive to mercaptopurine-induced myelosupression than the latter (Relling *et al*, 1999a,b, 2011, 2013; Karas-Kuzelicki *et al*, 2009 Peregud-Pogorzelski *et al*, 2011).

Both North American and European childhood ALL trials have shown that patients with lower TPMT activities and/or higher TGN levels have a lower relapse-risk (Lennard & Lilleyman, 1989; Schmeigelow *et al*, 1995; Balis *et al*, 1998; Relling *et al*, 1999b; Schmiegelow *et al*, 2009); *TPMT* heterozygotes have fewer relapses than those with a wild-type genotype (Schmiegelow *et al*, 2009; Lennard *et al*, 2015). In the Berlin-Frankfurt-Munster (BFM) 2000 study, patients heterozygous for *TPMT* low activity variant alleles had greater clearance of minimal residual disease (MRD) load, following the initial course of mercaptopurine (Stanulla *et al*, 2005). Minimal residual disease is the best measure of early response to chemotherapy and a sensitive and specific predictor of relapse risk in children with ALL in remission (Conter *et al*, 2010). Within the UK ALL97 and ALL97/99 trials the TPMT*1/*3A heterozygous patients had a better outcome than *TPMT* wild-type patients (Lennard *et al*, 2015). The aim of this study was to re-evaluate the impact of TPMT on treatment outcome in UKALL 2003, a trial with significantly improved outcomes compared to ALL97 (Vora *et al*, 2013).

## Methods

### Patients

The Medical Research Council (MRC) UK ALL 2003 (UKALL 2003) randomized control trial (registration number ISRCTN07355119) tested whether MRD-based risk stratification allows the intensity of therapy to be adapted to the risk of relapse. The trial had an add-on thiopurine biological study. The trial protocol was approved by the Scottish Multi-Centre Research Ethics Committee. Initially patients between 1 and 18 years were recruited from 45 centres in the UK and Ireland, but the upper age limit was gradually increased to 20 years from February 2006 and finally to 25 years by August 2007. Patient recruitment, National Cancer Institute (NCI) risk stratification and clinical high-risk groups have been previously described (Vora *et al*, 2013). Patients classified as clinical high risk (NCI re-classified cohorts, high-risk cytogenetics or slow morphological early response) were not eligible for MRD stratification.

The stratification of clinical standard and intermediate risk groups by bone-marrow MRD has been previously described (Vora *et al*, 2013). Briefly, MRD was measured after induction (day 29) and again after the recovery from consolidation but prior to the start of interim maintenance. Minimal residual disease low-risk patients were defined as those with no detectable disease and those patients who were MRD negative prior to interim maintenance. Indeterminate risk patients had detectable disease (<0·01% MRD = <10^−4^ leukaemia cells) prior to interim maintenance; this group also included those patients with no MRD measurement. High-risk patients had detectable disease (≥0·01%) at the end of induction. The treatment intensity randomizations of one or two delayed intensive blocks (reduced versus standard treatment) for low risk patients and standard treatment *versus* an intensive schedule for high-risk patients, have been previously described along with the complete chemotherapy regimens (Vora *et al*, 2013).

### Laboratory measurements

Minimal residual disease was measured by a standardized real-time quantitative polymerase chain reaction method for immunoglobulin and T-cell receptor antigen gene rearrangements within four UK laboratories participating in a European quality-assurance scheme (Flohr *et al*, 2008; Bruggemann *et al*, 2010). The quantitative range of the assay was 10^−4^ (0·01%): 1 leukaemic cell in 10 000 cells.

The Thiopurine Study protocol required a blood sample at disease diagnosis for classification of *TPMT* genotype prior to the start of mercaptopurine therapy. An additional blood sample (5 ml lithium heparin) was requested during remission maintenance chemotherapy for confirmation of *TPMT* genotype and measurement of mercaptopurine metabolites. The metabolite measurement was used as a reference sample for any future clinical thiopurine metabolism queries. The mercaptopurine chemotherapy blood sample was taken immediately before a monthly vincristine injection and requested at the earliest point in a maintenance cycle when patients were tolerating mercaptopurine at the standard protocol, or the maximum tolerated dose, for 2 weeks or more. If the patient's mercaptopurine dosage had been reduced or withdrawn the sample was taken on recovery of the cell counts during the next maintenance cycle. The primary thiopurine study was of *TPMT* genotype and mercaptopurine metabolite formation, if the chemotherapy blood sample was taken at least 2 months after the last red cell transfusion TPMT activity was also measured. Thiopurine metabolite concentrations, TPMT activities and *TPMT* genotypes were measured as previously described (Lennard *et al*, 2013). Thiopurine metabolite concentrations are measured as pmol/8 × 10^8^ red blood cells and stated in the text as pmol. TPMT activity is measured as units/ml packed red cells and stated in the text as units. The lower limit of detection and quantitation for the TGN metabolites were 6 and 30 pmol, respectively, and were 15 and 60 pmol, respectively, for the methyl-mercaptopurine nucleotide metabolites (MeMPNs; products of the TPMT reaction). The lower limit of detection and quantitation for TPMT activity was 0·75 units (= nil activity, TPMT deficiency). Blood samples were genotyped for TPMT*3A, TPMT*3B and TPMT*3C by amplification of exons 7 and 10 of the *TPMT* gene (TPMT*3A is an exon 7 and 10 double mutant); TPMT*2 and TPMT*9 were detected by sequencing exon 5 of the *TPMT* gene (Lennard *et al*, 2013). The TPMT *3 family and TPMT *2 low activity variant alleles account for ≥95% of variant *TPMT* alleles.

### Compliance with oral mercaptopurine chemotherapy

Clinicians forwarded additional blood samples for metabolite monitoring if non-compliance with oral mercaptopurine was suspected. Patients were suspected of non-compliance if blood counts remained high when the patient was prescribed prolonged mercaptopurine at the protocol standard, or higher, dose. Very low or absent mercaptopurine metabolite concentrations are strong indications of non-compliance.

### Statistics

Within ALL97 the event-free survival (EFS) for the TPMT*1/*3A heterozygote was far better than for TPMT*1/*3C children (*P* = 0·002). For the *a priori* power calculation we anticipated *TPMT* genotypes on 1845 patients over a six-year ALL2003 trial period. With these patient numbers we would expect approximately 20 heterozygote TPMT*1/*3C patients and 142 TPMT*1/*3A. To test the hypothesis that there is about a four-fold difference in event rates between these groups, as seen in ALL97, these numbers will give over 95% power to detect this with similar event rates (55% and 14% for TPMT*1/*3C and TPMT*1/*3A patients respectively), using a 2-sided *P*-value of 0·05. The EFS in ALL2003 is higher than for ALL97 (Vora *et al*, 2006, 2013). There is over 85% power to detect a similar difference but with decreased event rates of 40% and 10%, and over 80% for 32% and 8% for TPMT*1/*3C and TPMT*1/*3A patients, respectively.

The Anderson–Darling test was used to examine the fit of observations to a normal distribution. Metabolite values are stated as median and range. Differences between groups were compared by the Chi-square statistic, or the Mann–Whitney test. Outcome analysis was of EFS, with an event defined as time to relapse, secondary tumour or death, relapse-free survival (RFS), which was defined as time to relapse (excluding those patients who did not achieve a remission or died during initial induction or consolidation chemotherapy) and overall survival (OS), which was defined as time to death. Kaplan–Meier curves were calculated and comparisons between groups were performed by the log-rank statistic with stratification by age, gender and white blood cell (WBC) count at presentation. Cox regression multivariate analysis was used to test whether the effects of variables were independent. Statistical analyses were by sas, version 9·2 (SAS Marlow, Buckinghamshire, UK) or Minitab 16 (Minitab Ltd, Coventry, Warwickshire, UK). Follow- up was to 31 October 2013, with median follow-up (of those with *TPMT* genotypes) of 5 years 10 months, range 3 months to 10 years 1 month.

## Results

### Thiopurine analysis

**Fig. 1:**
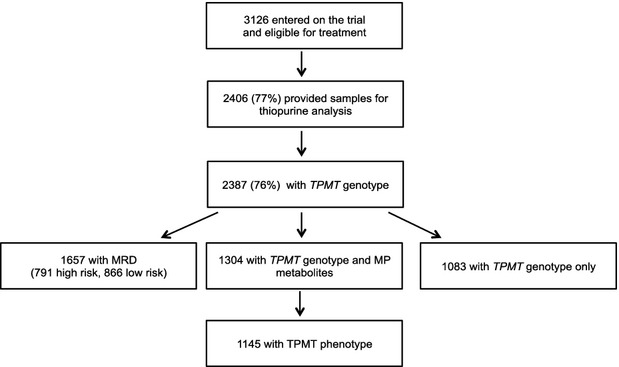
ALL2003 trial data profile. The numbers of individual patients providing blood samples for thiopurine analysis. TPMT, thiopurine methyltransferase; MP, mercaptopurine; MRD, minimal residual disease.

The patient numbers and samples available for analysis are summarized in Fig1. Significantly more blood samples were received for thiopurine analysis from younger patients and from less high-risk patients (Table I). Of the 2387 patients with a *TPMT* genotype available, 2190 were homozygous wild-type (TPMT*1/*1), 189 were heterozygous for low activity *TPMT* alleles (166 TPMT*1/*3A, 19 TPMT*1/*3C, 3 TPMT*1/*2 and 1 TPMT*1/*9) and 8 were TPMT-deficient (4 TPMT*3A/*3C, 3 TPMT*3A/*3A and 1 TPMT*2/*3A). Compared to the TPMT*3A allele there was an excess of the TPMT*3C allele in ethnic minorities (Chi-squared 10·57, *P* = 0·001; Table II).

**Table I:** Patient characteristics

Characteristic	Thiopurine data	No thiopurine data	Total	*P*-value
Sex				
Male	1369 (56·9%)	407 (56·5%)	1776	0·9
Female	1037 (43·1%)	313 (43·5%)	1350	
Age Group				
<10 years	1795 (74·6%)	492 (68·3%)	2287	0·0009
≥10 years	611 (25·4%)	228 (31·7%)	839	
WBC group				
<50 × 10^9^/l	1876 (78·0%)	559 (77·6%)	2435	0·9
≥50 × 10^9^/l	530 (22·0%)	161 (22·4%)	691	
NCI risk group				
Standard risk	1428 (59·4%)	388 (53·9%)	1816	0·009
High risk	978 (40·6%)	332 (46·1%)	1310	
CNS disease at diagnosis				
No	2365 (98·3%)	708 (98·3%)	3073	0·9
Yes	41 (1·7%)	12 (1·7%)	53	
Immunophenotype				
B/N	2102 (87·6%)	629 (87·5%)	2731	0·9
T	298 (12·4%)	90 (12·5%)	388	
Slow early response				
No	2142 (89·0%)	620 (86·1%)	2762	0·03
Yes	264 (11·0%)	100 (13·9%)	364	
MRD				
High	793 (33·3%)	237 (33·3%)	1030	0·0004
Indeterminate	710 (29·8%)	261 (36·7%)	971	
Low	877 (36·8%)	213 (30·0%)	1090	
Cytogenetic risk group (BCP ALL only)				
Good	1252 (62·7%)	332 (57·8%)	1584	0·04
Intermediate/Poor/High	745 (37·3%)	242 (42·2%)	987	
Treatment given				
A	1212 (50·4%)	326 (45·3%)	1538	0·009
B	652 (27·1%)	194 (26·9%)	846	
C	542 (22·5%)	200 (27·8%)	742	

WBC, white blood cell count (at diagnosis); NCI, National Cancer Institute; CNS, central nervous system; MRD, minimal residual disease; BCP ALL, B-cell precursor acute lymphoblastic leukaemia.

Comparing the *n* = 2406 patients who have thiopurine data (*TPMT* genotype and/or mercaptopurine metabolites) to the *n* = 720 with no data, there is some bias in the thiopurine dataset towards younger patients and those who are less high risk.

**Table II:** *TPMT* genotype by ethnicity

	*1/*1	*1/*2	*1/*3A	*1/*3C	*1/*9	*2/*3A	*3A/*3A	*3A/*3C	Unknown	Total
White	1790	2	152	12	1	1	3	4	567	2532
Asian	154	1	5	4					68	232
Black	53		1	1					19	74
Mediterranean	16								2	18
Middle Eastern	14		1						8	23
Mixed	60		2						11	73
Oriental	7		1						2	10
Other	22								6	28
Unknown	74		4	2					56	136
Total	2190	3	166	19	1	1	3	4	739	3126

Including the thiopurine methyltransferase (TPMT)-deficient children in the analysis there were 8 TPMT*3A alleles in ethnic minorities (160 in white patients) and 5 TPMT*3C alleles in ethnic minorities (16 in white patients), Chi-squared = 10·57 *P* = 0·001.

Of those patients categorized as MRD high or low risk 1657 had *TPMT* genotypes available: 791 high-risk MRD patients (728 *TPMT* wild-type and 63 heterozygotes) and 866 low-risk MRD patients (803 *TPMT* wild-type and 63 heterozygotes). There was no difference in the distribution of *TPMT* genotypes between the two MRD risk groups (Chi-squared 0·28, *P* = 0·597).

### Mercaptopurine metabolites

The median week for the reference blood sample was week 17 (range 11–162). Blood samples from patients on Regimen C skewed the data because these patients did not enter mercaptopurine maintenance until week 47; Regimen C patients had their reference sample at a median of week 66 (range 49–162), the median for Regimens A+B remained at week 17. In agreement with previous UK ALL trials (Lennard *et al*, 1990, 2013, 2015) mercaptopurine metabolite accumulation differed by genotype with *TPMT* wild-type patients accumulating lower concentrations of the TGN active metabolites than *TPMT* heterozygotes (Table III) or TPMT-deficient patients. The ALL2003 trial recommendation was for the TPMT-deficient patient to start mercaptopurine on a much-reduced dose (10% protocol), and titrate to the protocol target cell counts. Seven of the 8 TPMT-deficient children, identified pre-treatment, tolerated mercaptopurine dosages ranging from 8 to 26 mg/m^2^ (median 9 mg/m^2^) and TGN concentrations ranged from 970 to 2569 pmol (median 1329) in blood samples taken after a median of 8 weeks mercaptopurine (range 2 to >12 weeks). One child, who lacked a pre-treatment blood sample, was identified during maintenance chemotherapy with a history of repeated cytopenias and an inability to tolerate mercaptopurine; TGNs after 50 mg/m^2^ mercaptopurine for 6 weeks were 2347 pmol. TPMT-deficient patients do not accumulate MeMPNs.

**Table III:** Thiopurine methyltransferase genotype and metabolite formation

	Wild-type TPMT*1/*1	Heterozygous *TPMT*	Median difference (95%CI)
Patients	1187	109	
MP dose mg/m^2^	75 (7–232)	74 (17–93)	−1·0 (−2·0 to −0·001), *P* = 0·046
TGNs pmol	312 (0–1449)	751 (174–2597)	425 (372 to 482), *P* < 0·0001
MeMPNs pmol	14808 (0–83904)	4205 (0–37362)	−9055 (−10824 to −7350), *P* < 0·0001

A comparison of mercaptopurine (MP) metabolite formation in thiopurine methyltransferase (*TPMT*) wild-type and heterozygous patients. The MP dose is that tolerated at the time of metabolite measurement. TGNs, thioguanine nucleotides; MeMPNs, methylmercaptopurine nucleotides; CI, confidence interval. TGN and MeMPN units are pmol/8 × 10^8^ red cells. Values are given as median (range).

Additional blood samples were forwarded from some clinicians when patients were unduly sensitive to mercaptopurine or tolerating mercaptopurine prior to dose escalation. From 1304 patients we received 3514 blood samples taken during chemotherapy (median 2 samples per patient, range 1–72). Thirty-nine patients (3% of total cohort) had metabolite levels at the lower limit of detection or lacked measurable metabolites, six of these patients on multiple occasions. At the time of nil metabolites the mercaptopurine dosage ranged from 70 to 130 mg/m^2^ (median 76) for a median of 4 weeks (range 2–15); non-compliance with oral chemotherapy is the most logical explanation for these findings. There was no difference between the age range of those children with compliance problems and those without (median age 4·9 years, range 1·1–23·9).

### TPMT genotype-phenotype discordance

**Fig. 2:**
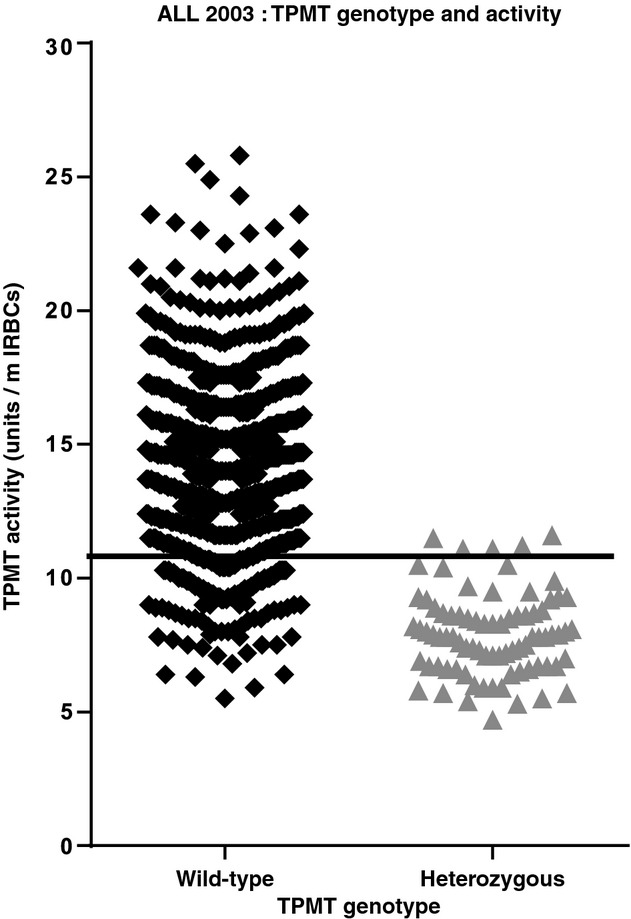
*TPMT* genotype-phenotype discordance, 61% of the intermediate TPMT activity cohort have a *TPMT* wild-type genotype. The solid line at TPMT 10·5 units indicates the nadir of the TPMT high and intermediate frequency distributions.

TPMT activity was available for 1045 patients who were *TPMT* wild-type, 92 heterozygotes and eight patients homozygous for variant alleles. The concordance in the homozygous variant allele cohort was 100%, all eight patients lacked TPMT activity. The break-point of the nadir of the TPMT ‘intermediate’ and ‘high’ frequency distributions was 10·5 units, a value determined by sensitivity and specificity analysis of the distribution of the *TPMT* heterozygous genotype over the TPMT activity range (Lennard *et al*, 2013). At 10·5 units the sensitivity for the detection of the *TPMT* heterozygous variant allele was 95% (specificity 87%). The specificity of 87% results in 13% of wild-type alleles in the intermediate activity cohort and a concordance of 39% (i.e. 61% of the intermediate activity cohort patients had a wild-type genotype). Within the intermediate activity cohort both the heterozygous variant allele (*n* = 87) and homozygous wild-type allele patients (*n* = 139) had similar TPMT activities ranging from 5·3–10·5 units and 5·5–10·5 units respectively (Fig2). The concordance in the high activity group (activity range 10·5–26·4 units) was 99·5% (906 wild-type alleles and 5 heterozygous variant alleles, the highest TPMT activity of a variant allele heterozygote was 11·6 units). As observed in ALL97 (Lennard *et al*, 2013), the median mercaptopurine metabolite concentrations measured in the intermediate activity *TPMT* wild-type genotype patients (317 pmol TGNs, 15 937 pmol MeMPNs) were similar to the concentrations measured in the high activity *TPMT* wild-type genotype patients (311 pmol TGNs, 14 380 pmol MeMPNs) and significantly different from the metabolite concentrations recorded for *TPMT* variant allele heterozygotes (747 pmol TGNs, 3407 pmol MeMPNs); intermediate activity heterozygous variant allele vs intermediate activity wild-type *TPMT* genotype patients, median difference 390 pmol TGNs (95% confidence interval, CI, 323–469, *P* < 0·0001) and −9995 pmol MeMPNs (95% CI −13446 to −6675, *P* < 0·0001).

### Clinical outcome

**Fig. 3:**
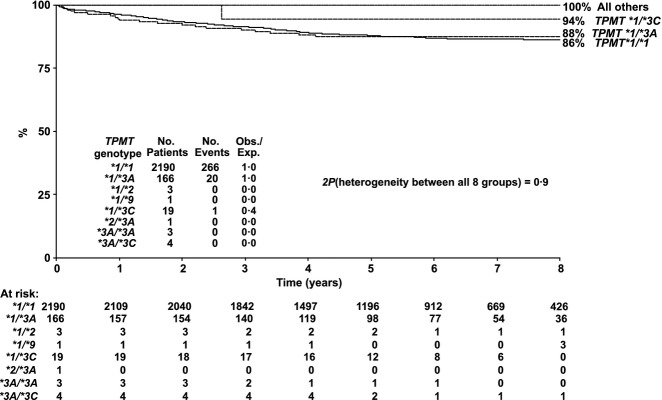
UKALL2003 – Event-free survival by TPMT genotype

There was no difference in OS, RFS or EFS between the *TPMT* genotypes. Five-year OS was 93% for TPMT*1/*1 and TPMT *1/*3A and 100% for all other *TPMT* genotypes. Five-year RFS was 92% for TPMT*1/*1 and TPMT *1/*3A, 94% for TPMT *1/*3C and 100% for all other TPMT genotypes. Five-year EFS was 88% for TPMT*1/*1 (*n* = 2190, 95% confidence interval, CI, 87–89%) and TPMT *1/*3A (*n* = 166, 95% CI 82–93%), 94% for TPMT *1/*3C (*n* = 19, 95% CI 84–100%) and 100% for all other *TPMT* genotypes (Fig3). The corresponding five-year EFS estimates for the previous ALL97 trial were 80% for TPMT*1/*1 (*n* = 1206, 95% CI 78–82%), 88% for TPMT *1/*3A (*n* = 99, 95% CI 81–94%) and 53% for TPMT *1/*3C (*n* = 17, 95% CI 29–77%) (Lennard *et al*, 2015).

For patients with metabolite data there was no difference in EFS between the nil metabolite, non-compliant, cohort and the rest of the group. There was no relationship between the trial reference metabolite concentrations and EFS when the data was analysed either as continuous variables or split into quartiles. There was no difference in EFS between the TPMT phenotype groups.

Although EFS differed by MRD risk group in ALL2003 there was no difference in EFS, with respect to *TPMT* genotype, within those risk groups (Table IV). In a multivariate Cox regression analysis the only significant factor affecting EFS in this subset of patients was MRD status (hazard ratio for high-risk MRD patients = 4·22, 95% CI 2·97–5·99, *P* < 0·0001).

**Table IV:** Event-free survival (EFS) and *TPMT* genotype within subgroups defined by MRD risk status

	MRD high-risk			MRD low-risk		
*TPMT* genotype	Events/Patients	O/E	5-year EFS (95% CI)	Events/Patients	O/E	5-year EFS (95% CI)
*1/*1	140/728	1·02	80·5% (77·6–83·4%)	37/803	0·97	95·4% (93·8–97·0%)
*1/*3A, *1/*2, *1/*9	9/58	0·80	83·5% (73·5–93·5%)	4/56	1·53	92·9% (86·2–99·6%)
*1/*3C	1/5	1·19	75·0% (32·5–100·0%)	0/7	0	100%
*P* (heterogeneity)			0·8			0·6

TPMT, thiopurine methyltransferase; MRD, minimal residual disease; O/E, Observed/Expected; CI, confidence interval. There was no heterogeneity with respect to *TPMT* genotype within the MRD subgroups defined by MRD risk status.

## Discussion

A major outcome of the ALL2003 trial was the much improved survival (5-year EFS 87%, 5-year OS 92%) compared to its predecessor ALL97 (5-year EFS 80%, 5-year OS 89%) (Vora *et al*, 2006, 2013). In contrast to ALL97, there was no difference in EFS between the *TPMT* genotypes. In both trials, the 5-year EFS for the TPMT*1/*3A cohort remained the same (88%), but the 5-year EFS for the TPMT*1/*1 cohort improved, from 80% in ALL97 (Lennard *et al*, 2015) to 86% in ALL2003. Importantly, the unexplained worse outcome for TPMT*1/*3C patients observed in ALL97 (5-year EFS 53%) was not observed in ALL2003 (5-year EFS 94%).

The chemotherapy backbone of ALL2003 was identical to the ALL97/99 phase of the ALL97 trial (Vora *et al*, 2006, 2013), but in ALL2003 all patients received dexamethasone instead of prednisolone and pegylated asparaginase instead of native *Escherichia Coli* asparaginase. Also, treatment intensity was stratified by MRD response. MRD low risk patients fared much better than the high-risk patients (5-year EFS 95% versus 80%, respectively). Overall there was no difference in survival, with respect to *TPMT* genotype, within the two MRD risk groups. The BFM2000 trial used a two time-point assessment protocol, measuring the disease load before (day 33) and after (day 78) the initial course of mercaptopurine chemotherapy (Conter *et al*, 2010), to study the clearance of disease with respect to *TPMT* genotype: patients heterozygous for variant *TPMT* alleles had an increased clearance of disease, a lower rate of MRD positivity (Stanulla *et al*, 2005). Minimal residual disease was not monitored at two points in UK ALL2003 thus; the association of *TPMT* genotype with MRD response following thiopurine exposure could not be investigated.

This study confirms previous observations on non-compliance with oral chemotherapy within the UK ALL protocols (Lennard *et al*, 1995, 2015) and illustrates the usefulness of metabolite monitoring in the identification of the non-compliant patient. A complete lack of mercaptopurine metabolites when taking prolonged high doses, as observed in 3% of patients in this study, is a strong indication of non-compliance with oral chemotherapy.

In a study of adolescents with ALL, improved compliance with mercaptopurine was associated with parental supervision of tablet taking (Malbasa *et al*, 2007). Evaluation of mercaptopurine non-compliance in ALL children by mercaptopurine metabolite monitoring coupled with structured interviews has indicated that medication non-compliance rates could be as high as 26% (Hawwa *et al*, 2009) with a lower non-compliance associated with adverse socioeconomic factors (De Oliveira *et al*, 2004). Evaluation of mercaptopurine non-compliance by an event-monitoring cap on the medication bottle associated non-compliance with ethnicity and an increased relapse risk; the association with ethnicity was linked to a lower socioeconomic status (Bhatia *et al*, 2012). The previously reported worse outcome for TPMT*1/*3C patients (Lennard *et al*, 2015) may have been influenced by the increased frequency of this *TPMT* allele in ethnic minorities.

This study has also confirmed the previously reported discordance between *TPMT* genotype and phenotype, which has been mainly attributed to the undue influence of the disease process and chemotherapy on red blood cell TPMT enzyme activity (Lennard *et al*, 2013). In this patient group, TPMT activity should not be used to predict *TPMT* heterozygosity. An improvement in outcome for *TPMT* wild-type patients has closed the EFS gap with TPMT*1/*3A heterozygous patients. However, the EFS for the TPMT*1/*3A heterozygous cohort has large confidence intervals and so a small difference in outcome between the *TPMT* wild-type and TPMT*1/*3A patients cannot be excluded. The improvement is likely to be due to a combination of better risk stratification and use of dexamethasone and pegylated asparaginase throughout treatment. Treatment intensification can influence the subsequent response to mercaptopurine maintenance chemotherapy. This was initially reported in MRC UKALL X (Chessells *et al*, 1997) and observed subsequent to asparaginase therapy in the United States Dana Farber Cancer Institute protocols (Merryman *et al*, 2012) and could contribute to the improved outcome for the *TPMT* wild-type patients.
